# Generation of myocyte agonal Ca^2+^ waves and contraction bands in perfused rat hearts following irreversible membrane permeabilisation

**DOI:** 10.1038/s41598-023-27807-w

**Published:** 2023-01-16

**Authors:** Yuma Morishita, Shoko Tamura, Kentaro Mochizuki, Yoshinori Harada, Tetsuro Takamatsu, Hajime Hosoi, Hideo Tanaka

**Affiliations:** 1 Department of Pathology and Cell Regulation, Kyoto Prefectural University of Medicine, Graduate School of Medical Science, Kyoto, 602-8566 Japan; 2 Department of Pediatrics, Kyoto Prefectural University of Medicine, Graduate School of Medical Science, Kyoto, 602-8566 Japan; 3 Department of Medical Photonics, Kyoto Prefectural University of Medicine, Kyoto, 602-8566 Japan

**Keywords:** Physiology, Cardiology, Pathogenesis

## Abstract

Although irreversible cardiomyocyte injury provokes intracellular Ca^2+^ ([Ca^2+^]_i_) overload, the underlying dynamics of this response and its effects on cellular morphology remain unknown. We therefore visualised rapid-scanning confocal fluo4-[Ca^2+^]_i_ dynamics and morphology of cardiomyocytes in Langendorff-perfused rat hearts following saponin-membrane permeabilisation. Our data demonstrate that 0.4% saponin-treated myocytes immediately exhibited high-frequency Ca^2+^ waves (131.3 waves/min/cell) with asynchronous, oscillatory contractions having a mean propagation velocity of 117.8 μm/s. These waves slowly decreased in frequency, developed a prolonged decay phase, and disappeared in 10 min resulting in high-static, fluo4-fluorescence intensity. The myocytes showing these waves displayed contraction bands, i.e., band-like actin-fibre aggregates with disruption of sarcomeric α-actinin. The contraction bands were not attenuated by the abolition of Ca^2+^ waves under pretreatment with ryanodine plus thapsigargin, but were partially attenuated by the calpain inhibitor MDL28170, while mechanical arrest of the myocytes by 2,3-butanedione monoxime completely attenuated contraction-band formation. The depletion of adenosine 5′-triphosphate by the mitochondrial electron uncoupler carbonyl cyanide 4-trifluoromethoxy phenylhydrazone also attenuated Ca^2+^ waves and contraction bands. Overall, saponin-induced myocyte [Ca^2+^]_i_ overload provokes agonal Ca^2+^ waves and contraction bands. Contraction bands are not the direct consequence of the waves but are caused by cross-bridge interactions of the myocytes under calpain-mediated proteolysis.

## Introduction

Intracellular Ca^2+^ ([Ca^2+^]_i_) overload is a cardinal feature of cardiomyocyte injury, and its progression to an irreversible state leads to cell death^1, 2^. The myocytes suffering from [Ca^2+^]_i_ overload present with Ca^2+^ waves, i.e., spontaneous propagating rises in the [Ca^2+^]_i_ concentration, which accompany localised contractions^3–6^. Our previous work demonstrated that myocytes in Langendorff-perfused rat hearts exhibit three different patterns of Ca^2+^ waves, with each pattern depending on the degree of [Ca^2+^]_i_ overload^7^. Of these, extremely high-frequency Ca^2+^ waves, called “agonal waves”, have been regarded as the representative [Ca^2+^]_i_ dynamics in irreversibly injured myocytes, e.g., myocytes subjected to cryoinjury^8^ or Ca^2+^-paradox injury^9^. We have also observed that irreversible membrane injury inflicted on cardiomyocytes by saponin initiates extremely high-frequency Ca^2+^ waves, which are comparable to those induced by Ca^2+^-paradox injury^9^. However, the precise spatiotemporal [Ca^2+^]_i_ dynamics under the irreversible [Ca^2+^]_i_ overload and their effect on the cellular morphology leading to their eventual cell death remain unknown. To address these issues, we sought to visualise the myocyte [Ca^2+^]_i_ dynamics induced by saponin in the Langendorff-perfused rat heart. We chose saponin because this chemical simply permeabilises the sarcolemmal membrane^10^, and the resultant entry of Ca^2+^ through the membrane pores provides definitive, reproducible induction of irreversible [Ca^2+^]_i_ overload compared with other injury models. For example, the cryoinjured model exhibits diverse and complex myocyte [Ca^2+^]_i_ dynamics, including high-frequency Ca^2+^ waves with or without diminution by Ca^2+^ transients and high-static [Ca^2+^]_i_, an indication of cell death^8^; therefore, their morphological relevance is minimal. The Ca^2+^-paradox model, which has been widely studied as an alternative model of ischaemia‒reperfusion injury, shows detachment of cell‒cell junctions with hypercontracture of myocytes due to cadherin inhibition induced by Ca^2+^ depletion^9^.

Here, we postulate that saponin-induced Ca^2+^ waves contribute to the formation of contraction bands*,* a morphological pattern associated with reperfusion injuries following ischaemia^11, 12^. We evaluated this theory using high-speed imaging of the subepicardial myocyte [Ca^2+^]_i_ dynamics in saponin-perfused rat hearts via rapid-scanning confocal microscopy together with confocal fluorescence imaging of myocyte morphology to address whether and how the Ca^2+^ waves contribute to the formation of contraction bands. Some of the results have been published as an abstract^13^.

## Results

### Saponin-induced membrane permeabilisation generates Ca^2+^ waves

**Figure 1:**
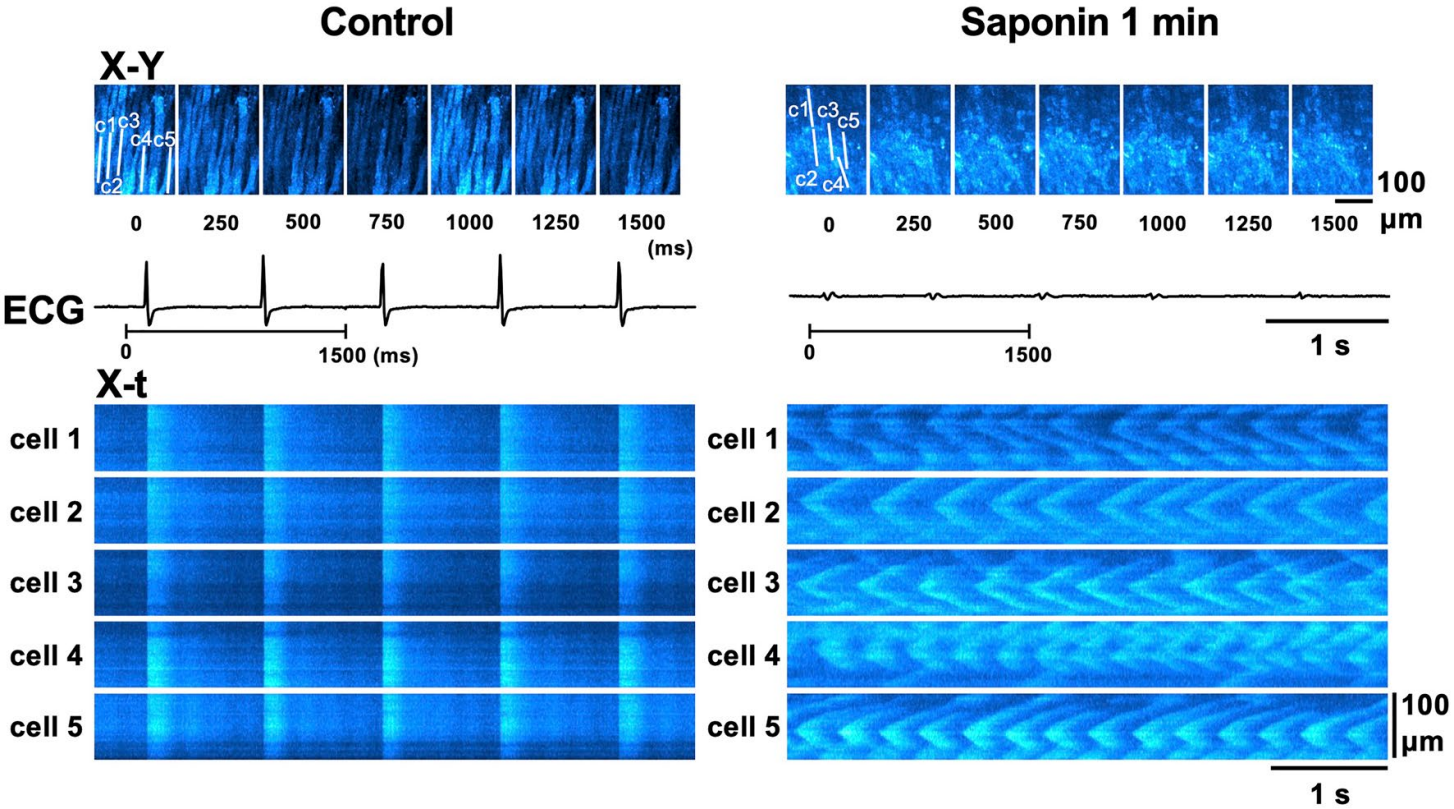
Development of high-frequency Ca^2+^ waves in response to saponin treatment. Sequential X–Y images (top panels, taken every 250 ms) of the subepicardial regions in the left ventricular myocardium over a 1500-ms period as indicated by the horizontal lines below the electrocardiogram (ECG) under the control conditions (left) and 1 min after the introduction of the saponin treatment (right). The X-t images (lower panels) for the fluo4-fluorescence intensity of five different cardiomyocytes (cells 1–5) when evaluated along the c1–c5 lines (100 μm) identified in the top panels.

Figure 1 shows the representative [Ca^2+^]_i_ dynamics of the subepicardial myocardium before and during saponin perfusion. In the absence of saponin, individual myocytes exhibited spatiotemporally uniform Ca^2+^ transients during systole and no abnormal increases in [Ca^2+^]_i_ concentration during diastole as shown in the sequential X–Y fluo4-fluorescence images and corresponding X-t images (see Supplementary Fig. 1a for analytical method) for five different myocytes (cells 1–5). However, just 1 min into saponin perfusion the individual myocytes exhibited highly frequent, repetitive Ca^2+^ waves with oscillatory contractions occurring asynchronously among the myocytes (Fig. 1 and Supplementary Video 1). These high-frequency Ca^2+^ waves were also asynchronous to the basic heart rhythm, where the QRS complex in the electrocardiogram (ECG) progressively decreased in amplitude and widened, eventually leading to ventricular asystole. In addition, the myocytes producing the high-frequency Ca^2+^ waves were unresponsive to electrical stimulation of the heart, and the properties of the Ca^2+^ waves were not influenced by external electrical stimulation (Supplementary Fig. 2). The Ca^2+^ waves propagated uni- or bidirectionally within the individual myocytes at a constant propagation velocity (V_prop_) of 117.8 ± 23.2 μm/s (n = 60 waves), revealing fixed patterns of intracellular propagation (see Supplementary Fig. 1b for analytical method). These waves propagated throughout the individual myocytes, but did not extend to their neighbouring cells.

We confirmed the saponin-induced membrane permeabilisation of the myocardium by confocal fluorescence images of the membrane dye di-4-ANEPPS and the nuclear dye propidium iodide (PI) in the heart. In the absence of saponin, the cell membrane and T-tubules were clearly visualised by di-4-ANEPPS but no nuclear fluorescence of PI was identified. In contrast, after saponin treatment, the cell membrane and T-tubules became ambiguous, and the di-4-ANEPPS dye was redistributed into the cytosol and the PI fluorescence into the nuclei (Supplementary Fig. 3).

### Sequential changes in the saponin-induced Ca^2+^ waves

**Figure 2:**
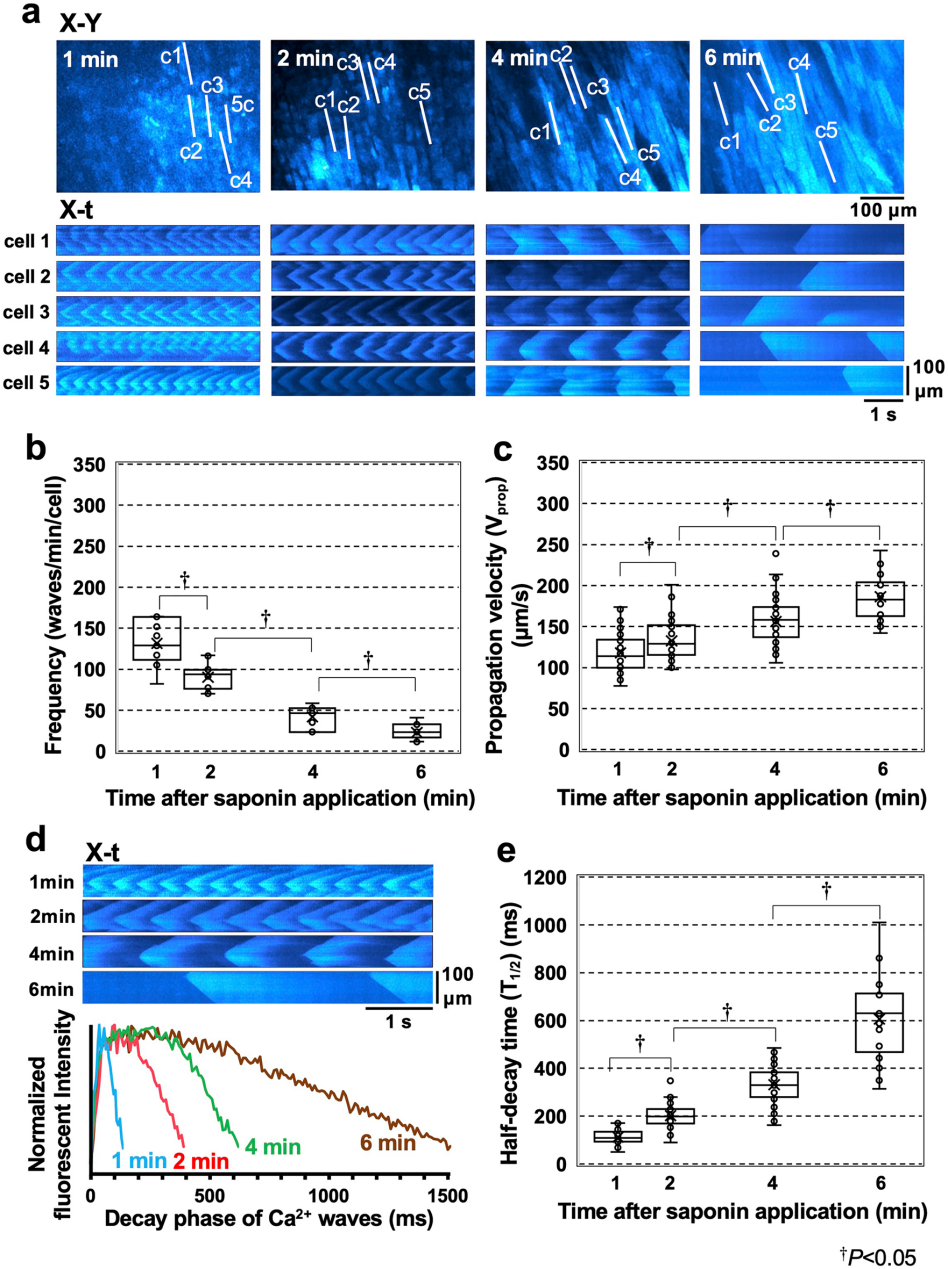
Sequential changes in the nature of the high-frequency Ca^2+^ waves induced by saponin treatment. (**a**) X–Y images (upper panel) and corresponding X-t images (lower panel) for each of the five different cardiomyocytes (cells 1–5) scanned along the lines (c1–c5) identified in the X–Y images over a 6 min period. The sequential changes in the frequency (**b**) and propagation velocity (V_prop_) (**c**) of the Ca^2+^ waves in these cells are represented as box plots. (**d**) Representative X-t images for each of the Ca^2+^ waves (upper panel) and the superimposed plot profiles of the wave decay patterns at 1, 2, 4, and 6 min after saponin treatment (lower panel). Each plot profile is shown as an averaged value of the profiles obtained from five different horizontal lines in each X-t image. (**e**) Sequential changes in the half-decay time (T_1/2_) of the Ca^2+^ waves. Statistical analyses were performed using the Kruskal‒Wallis test followed by the Steel‒Dwass test. ^†^*P* < 0.05.

The continued application of saponin resulted in a gradual decrease in the frequency of the Ca^2+^ waves, which nearly disappeared within 10 min of exposure, during which time the affected myocytes exhibited sporadic termination of Ca^2+^ waves showing high, static fluo4-fluorescence intensity, indicating progressive [Ca^2+^]_i_ overload (Fig. 2a and Supplementary Video 2). Quantitative evaluation revealed that the frequency of the Ca^2+^ waves decreased from 131.3 ± 25.1 waves/min/cell at 1 min of exposure to 23.0 ± 9.3 waves/min/cell at 6 min post induction (n = 60 cells) (Fig. 2b). On the other hand, the V_prop_ value for these waves increased with time from 117.8 ± 23.2 μm/s at 1 min post exposure to 186.2 ± 26.3 μm/s at 6 min post-exposure (n = 196 waves) (Fig. 2c), suggesting that this value was inversely correlated with the frequency of Ca^2+^ waves (Supplementary Fig. 4a). The progressive reduction in Ca^2+^-wave frequency was accompanied by a prolongation of their decay time course, as shown in the representative X-t images and the superimposed plot profiles for each wave’s fluorescence intensity (Fig. 2d) and half-decay time (T_1/2_) (n = 185 waves) (Fig. 2e, see Supplementary Fig. 1c for analytical method). In practice, there was an inverse relationship between the frequency and T_1/2_ of the Ca^2+^ waves. Thus, the lower the frequency was, the longer the T_1/2_ of the wave with remarkable prolongation at 6 min (Supplementary Fig. 4b). The progressive changes in the properties of these Ca^2+^ waves were analogously observed even by a single short-term application for 2 min and subsequent washout of saponin with time courses of the changes in frequency, V_prop_ and T_1/2_ of the waves (n = 3 hearts) similar to those under continued saponin perfusion (Supplementary Fig. 5), indicating that the saponin-induced membrane permeabilisation is irreversible.

The sequential changes in the Ca^2+^ waves indicate progressive reductions in both Ca^2+^ release from the sarcoplasmic reticulum (SR) and the reuptake of Ca^2+^ into the SR via Ca^2+^-ATPase (SERCA). This is because [Ca^2+^]_i_ overload following membrane permeabilisation can lead to the depletion of cellular ATP reserves by opening of the mitochondrial permeability transition pore (mPTP)^14^. Given this, we evaluated sequential changes in the mitochondrial membrane potential using a tetramethylrhodamine methyl ester (TMRM) fluorescence-based assay^15, 16^ (Supplementary Fig. 6). The assays revealed that the intensity of the TMRM fluorescence was maintained for over 15 min in the absence of saponin (n = 3 hearts) and was lost in almost 50% of the myocytes within 6 min of saponin perfusion, eventually disappearing in nearly all of the cells at 12 min (n = 3 hearts). Of note, saponin induced the loss of TMRM fluorescence intensity not uniformly but sporadically in individual myocytes, indicating that the effect was not due to the nonspecific leakage of TMRM from the mitochondria but to the loss of mitochondrial membrane potentials in agreement with previous demonstrations on the ischaemic myocardium^15, 16^.

### Saponin induction of contraction band formation and its responses to pharmacological interventions

**Figure 3:**
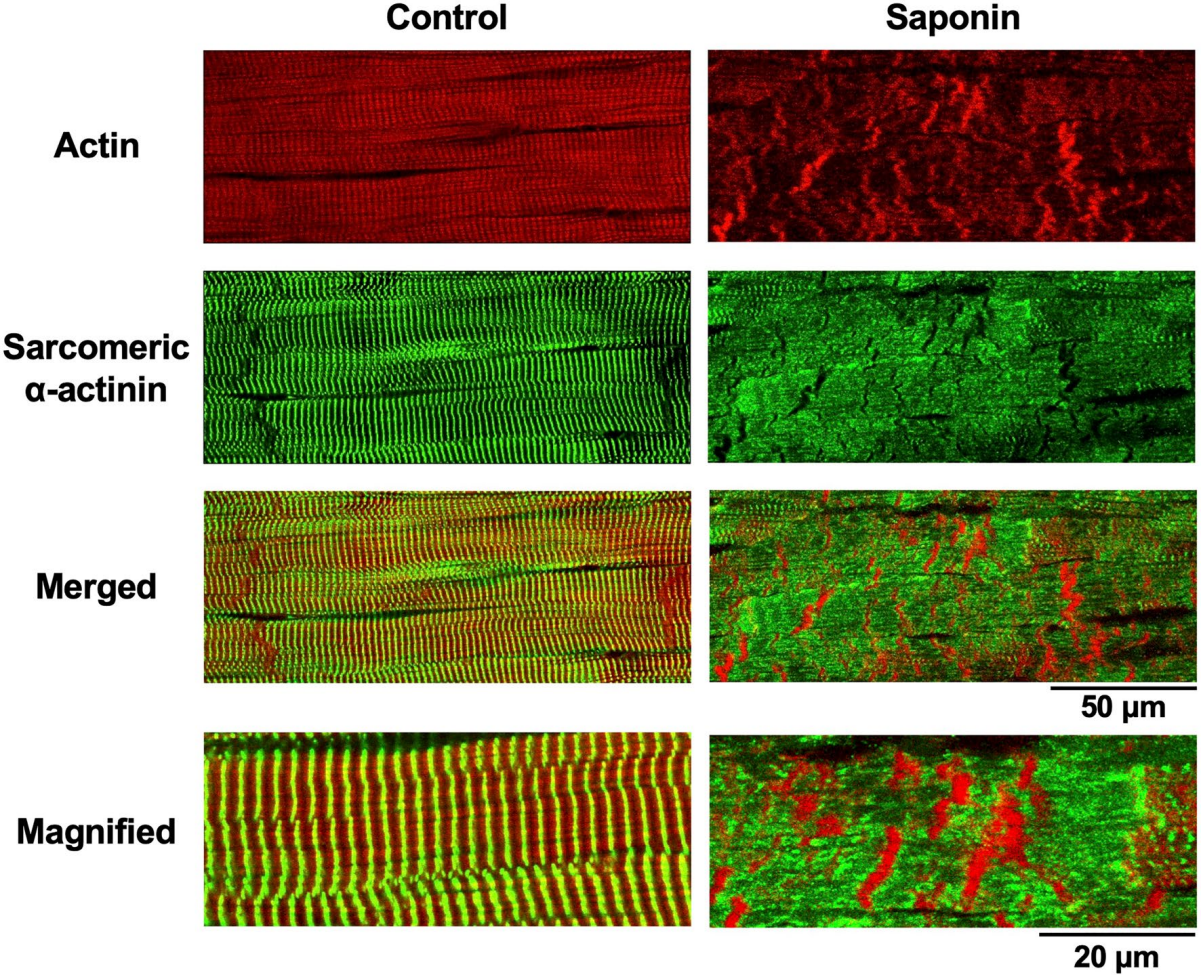
Contraction-band formation in saponin-treated myocardial samples as revealed by confocal fluorescence imaging of actin and α-actinin. Images present the high-affinity actin probe phalloidin (**red**), sarcomeric α-actinin immunohistochemistry (**green**), and merged images in the absence (**left**) and presence of saponin (**right**). Magnified images of the merged evaluations are shown on the bottom. Note that saponin induces contraction-band formation, increasing actin aggregation and disrupting α-actinin.

**Figure 4:**
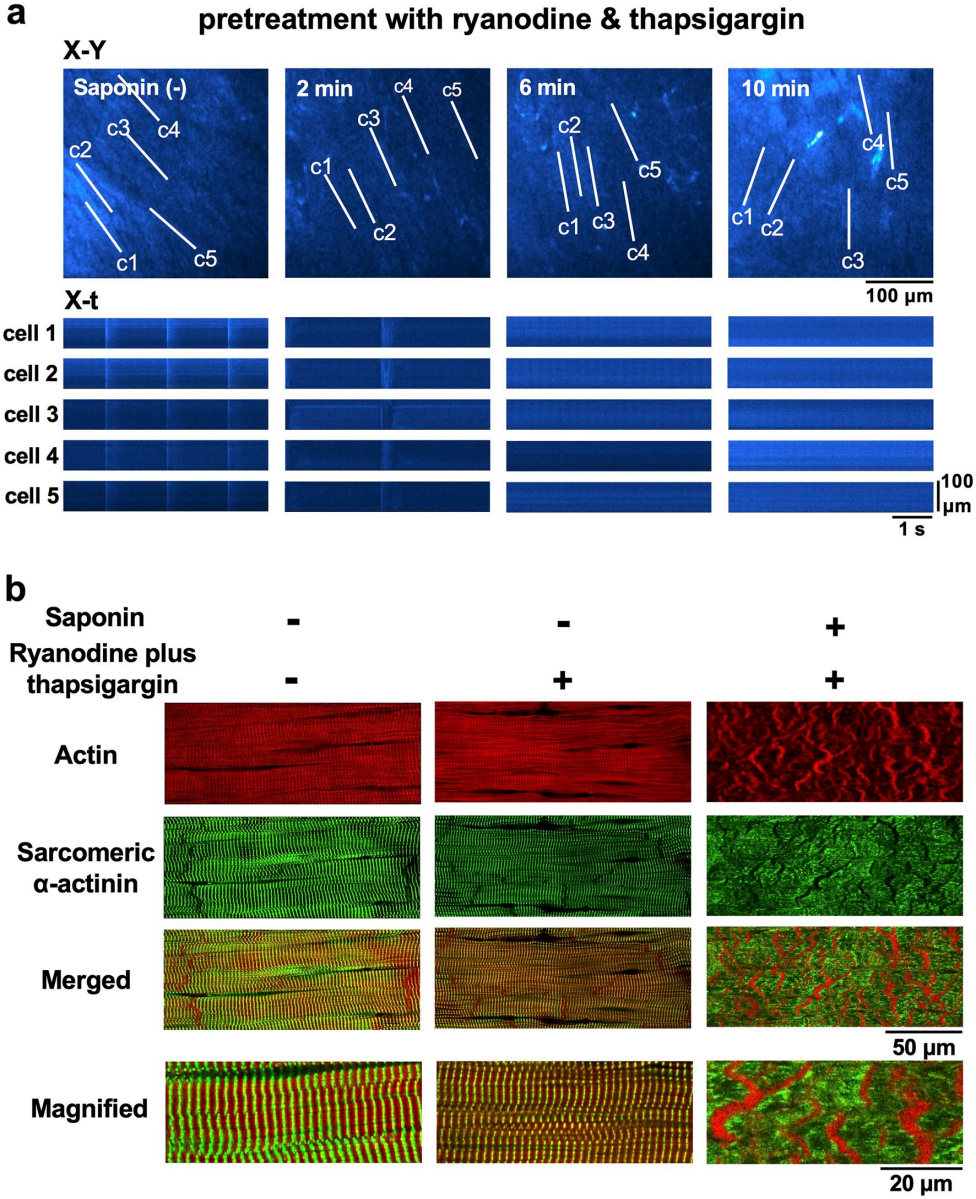
Changes in the saponin-induced contraction bands under depletion of Ca^2+^ released from the SR by ryanodine (1 μM) and thapsigargin (5 μM). (**a**) The X–Y and X-t fluo4-fluorescence images before and 2 min, 4 min, and 6 min after commencement of saponin perfusion. Note the absence of Ca^2+^ waves before and after saponin application. (**b**) Fluorescence images of the subepicardial myocardium in the absence of saponin with (**middle**) and without (**left**) ryanodine (1 μM) and thapsigargin (5 μM). Fluorescence images of the saponin-induced contraction bands under combined pretreatment with ryanodine and thapsigargin (**right**).

Saponin treatment resulted in an abundance of myocytes presenting with contraction bands, which were characterised by band-like aggregations of their regular actin fibres and clear disruptions in their α-actinin arrangements (Fig. 3). Surprisingly, the attenuation of Ca^2+^ release from the SR after the combined application of ryanodine (1 μM) and thapsigargin (5 μM)^17^, under which conditions myocytes barely showed Ca^2+^ transients or Ca^2+^ waves (Fig. 4a), failed to attenuate the production of the saponin-induced contraction bands (Fig. 4b).

**Figure 5:**
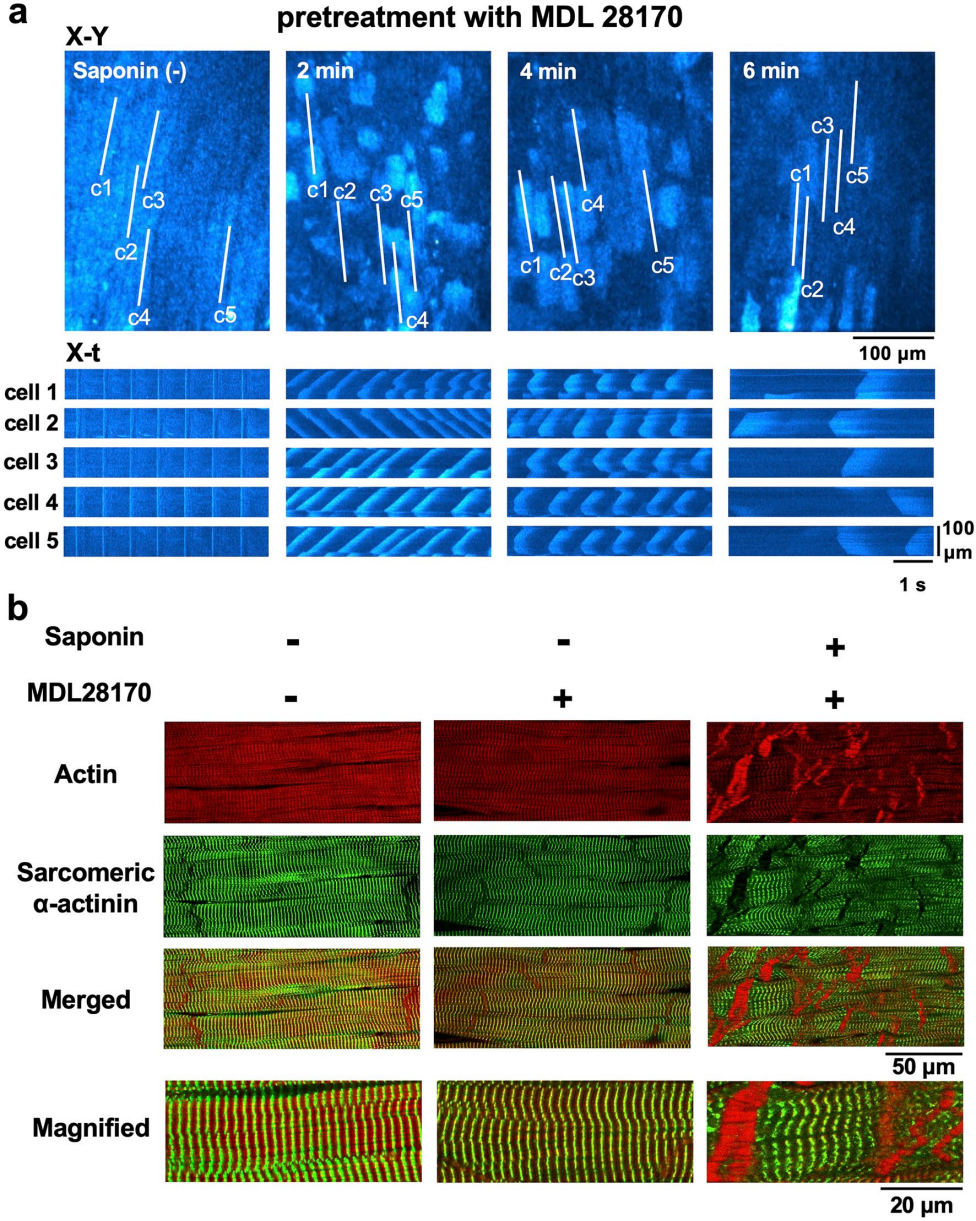
Role of calpain in the production of saponin-induced contraction bands. (**a**) Sequential changes in the saponin-induced Ca^2+^ waves following pretreatment with MDL28170 at 100 μM. (**b**) Confocal fluorescence images of the myocardium in the absence (**left**) and presence (**middle**) of MDL28170 at 100 μM. Confocal fluorescence images of saponin-induced contraction bands in the presence of MDL28170 (**right**). Note that the saponin fails to disrupt α-actinin under pretreatment with MDL28170.

Given this, we evaluated whether calpain, a protease activated during [Ca^2+^]_i_ overload^18^, contributes to the formation of the contraction bands. Under treatment with the calpain inhibitor MDL28170^19^ at 100 μM, the properties of the saponin-induced Ca^2+^ waves were not different from those in the absence of this inhibitor (Fig. 5a). Pretreatment with MDL28170 partially attenuated the saponin-induced derangements of cell structure: regular alignments of the α-actinin in these cells were preserved even when saponin was applied, whereas the aggregation of actin fibres was not attenuated by saponin (Fig. 5b).

**Figure 6:**
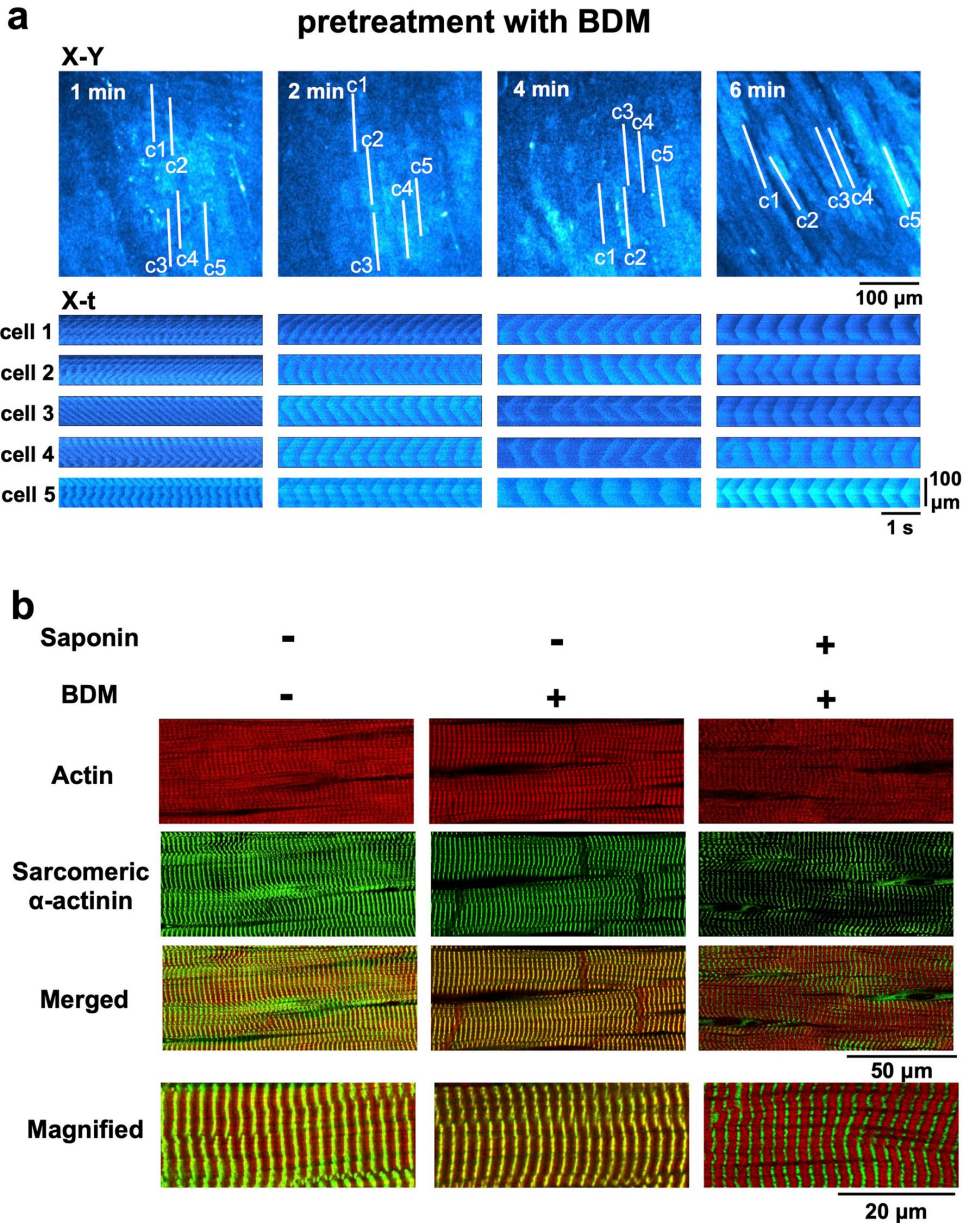
Prevention of saponin-induced contraction-band formation under pretreatment with 2,3-butanedione monoxime (BDM). (**a**) Sequential changes in saponin-induced Ca^2+^ waves in the presence of BDM (20 mM). X–Y images (upper) and the corresponding X-t images (cells 1–5) scanned along the c1–c5 lines (100 μm) derived from the individual myocytes identified in the X–Y images. (**b**) Images include myocytes under control conditions (**left**), in the presence of 20 mM BDM (**middle**), and under saponin treatment in the presence of BDM (**right**).

Mechanical cardiac arrest, induced by 2,3-butanedione monoxime (BDM) at 20 mM^20–23^ may attenuate contraction band formation. In the presence of BDM, saponin perfusion still induced the generation of agonal waves (Fig. 6a). Quantitatively, the waves (n = 60 waves) showed a frequency of 253.8 ± 40.0 waves/min/cell (n = 15 cells) and a V_prop_ of 118.7 ± 16.8 μm/s (Supplementary Fig. 7a,b). Of note, in the BDM treated hearts, the saponin-induced Ca^2+^ waves were significantly more frequent, with a higher V_prop_ and shorter T_1/2_ than their untreated counterparts (Supplementary Fig. 7b,c). Despite the persistence of agonal waves in the presence of BDM, saponin induced no changes in the alignment of actin or α-actinin under treatment with BDM (Fig. 6b).

**Figure 7:**
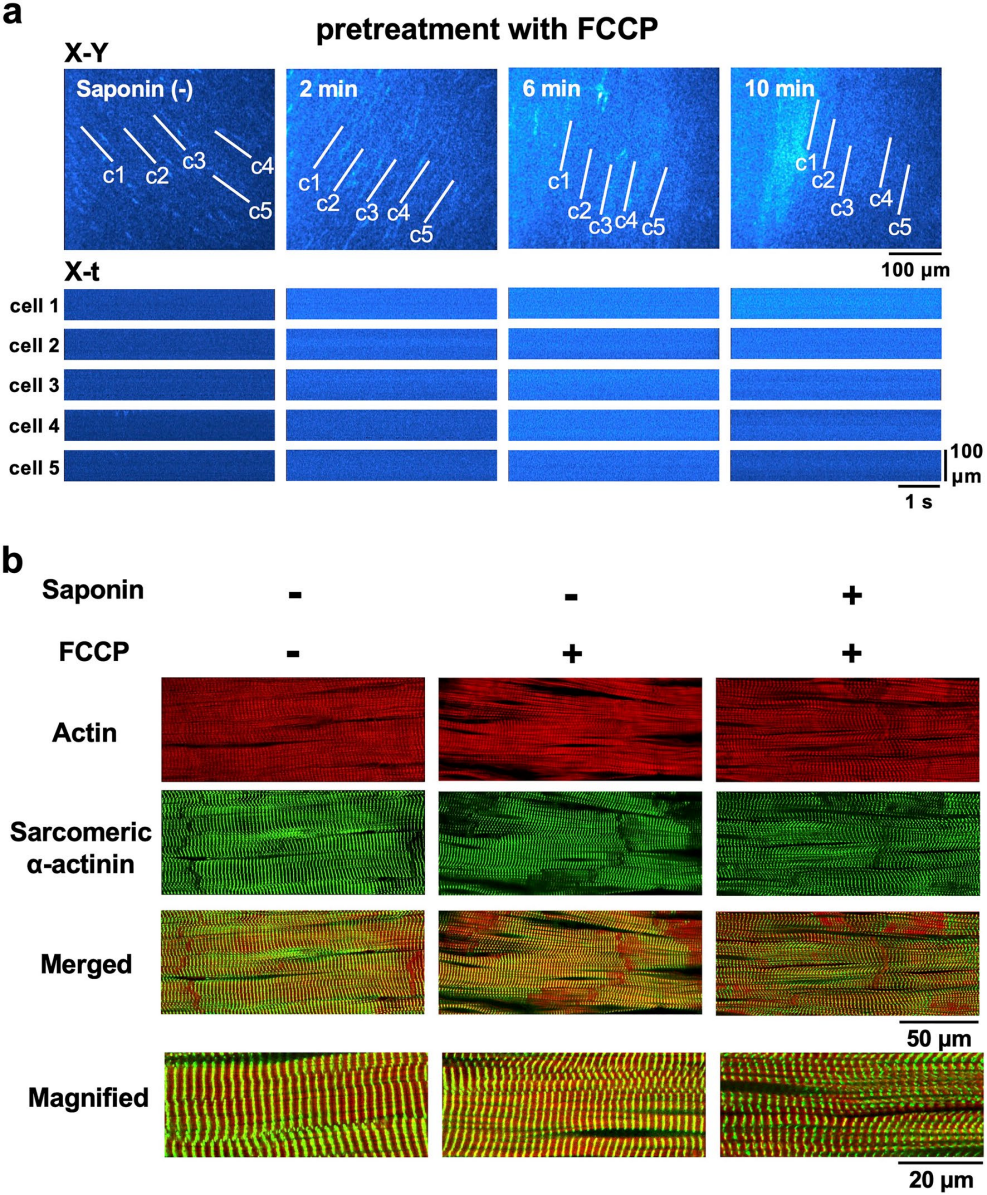
Saponin attenuates contraction-band formation in the myocardium treated with the mitochondrial uncoupling agent FCCP. (**a**) Fluo4-fluorescence images of the myocytes (cell 1–cell 5) before and 2-, 6-, and 10-min after the addition of saponin in the presence of FCCP at 10 μM. (**b**) Confocal fluorescence images of both actin filaments and α-actinin in the subepicardial myocardium of the control (**left**), in the presence of 10 μM FCCP (**middle**), and saponin treatment in the presence of FCCP (**right**).

When the mitochondrial electron transport chain was uncoupled by carbonyl cyanide 4-trifluoromethoxy phenylhydrazone (FCCP) at 10 μM, which depletes intracellular ATP^24^, myocytes showed no Ca^2+^transients or Ca^2+^ waves (Fig. 7a), consistent with the outcomes of intracellular ATP depletion. Under these conditions, the heart was rendered totally quiescent with no discernible mechanical contractions; saponin thus failed to induce contraction bands and there were no changes in either the actin or α-actinin arrangements in these cells, resulting in attenuation of contraction-band formation (Fig. 7b).

## Discussion

The present study demonstrates the high-frequency Ca^2+^ waves and coexisting contraction bands within the myocytes of perfused rat hearts following saponin-permeabilised membrane injury. The novel points of this study are as follows: (i) the high-frequency Ca^2+^ waves provoked by irreversible injury show progressive alterations during the progress toward cell death, and (ii) the simultaneous contraction band formation is not the direct consequence of the Ca^2+^ waves but might be mediated by cross-bridge interactions and calpain-mediated proteolysis as a result of [Ca^2+^]_i_ overload.

The observed Ca^2+^ waves here were similar to those of our previous studies, which allowed us to identify these responses as “agonal” Ca^2+^ waves^7, 8^. These waves present as asynchronous, ripple-like wavefronts with oscillatory contractions within individual myocytes induced by an irreversible [Ca^2+^]_i_ overload. These properties of the Ca^2+^ waves are likely to be due to the enhanced release of Ca^2+^ from the SR and the SERCA-mediated reuptake of Ca^2+^ into the SR in an oscillatory fashion^25^ and the unresponsiveness to electrical stimulation might be due to gap-junctional uncoupling via [Ca^2+^]_i_ overload^26, 27^.

Our data revealed that the properties of the saponin-induced agonal Ca^2+^ waves were not static but changed with time. Our understanding is that the initiation and subsequent alterations of the behaviours of agonal waves depend on both the concentrations of saponin and [Ca^2+^]_i_ and the time after saponin membrane permeabilisation: the higher the concentrations of saponin and [Ca^2+^]_i_ are, the more rapidly the agonal waves and subsequent transition to cell death develop showing high-static fluo4 fluorescence intensity.

The reduction in the wave frequency observed in the present study can be explained by progressive changes in the intracellular environment and the potential disruption of both SR and SERCA following calpain-mediated proteolysis^28–30^. Such alterations are likely to produce gradual declines in [Ca^2+^]_i_ cycling and possible depletion of ATP facilitated by the opening of the mPTP^14^ in response to progressive [Ca^2+^]_i_ overload. In practice, [Ca^2+^]_i_ overload in the saponin-treated myocytes was accompanied by progressive mitochondrial damage as indicated by the progressive, sporadic loss of TMRM fluorescence intensity in individual myocytes (Supplementary Fig. 6), an indication of the loss of the mitochondrial membrane potential^15, 16^, not of nonspecific diffusion of the probe via the permeabilised membrane.

Unexpectedly, the gradual increase in V_prop_ in response to the decrease in the wave frequency is contradictory to the principal properties of the Ca^2+^ wave, i.e., the greater the [Ca^2+^]_i_ overload is, the higher the frequency and V_prop_ given that both the frequency and V_prop_ depend on [Ca^2+^]_i_ concentration^31^. In this regard, we assume that prolonged [Ca^2+^]_i_ overload results in a limit in the buffering capacity of the [Ca^2+^]_i_ following excessive binding of Ca^2+^ to its intracellular buffers, e.g., sarcolemmal membrane, troponins, calmodulin, and the mitochondria^32, 33^. This saturation would then promote intracellular propagation of the waves, potentially increasing V_prop_ despite decreasing wave frequency. In addition, this reduced [Ca^2+^]_i_ buffering capacity would also augment [Ca^2+^]_i_ overload, which may well promote degradation of the intracellular organelles and cell structures by calpain^18^ and accelerate ATP depletion via the opening of the mPTP^14^.

Saponin-induced membrane permeabilisation progressively lengthened the half-decay time (T_1/2_) of these agonal waves. In this regard, the reduction in wave frequency may result in the lengthening of the Ca^2+^ restitution kinetics in the SR^34^, but this would not adequately account for the extensive prolongation of T_1/2_ observed in this study. Instead, we assume that the dissipation of cellular ATP may be responsible for these increased T_1/2_ values, as they markedly increased after 6 min of saponin exposure (Supplementary Fig. 4b), at which time the fluorescence intensity of TMRM was significantly reduced indicating opening of the mPTP. The decreases in the ATP content of these cells may well lead to inhibition of SERCA, which would slow the decay and reduce the frequency of the Ca^2+^ waves^35^. One may consider that saponin progressively promotes the leakage of ATP through the permeabilised membrane. However, considering that the T_1/2_ of the Ca^2+^ wave was not so quickly prolonged by saponin but remained relatively consistent, especially in the presence of BDM (Supplementary Fig. 7c), we assume that the amount of ATP, if decreased by diffusion, would not undergo much leakage in the heart by saponin membrane permeabilisation.

Our data also suggest that the production of the saponin-induced contraction bands in these tissues was not a direct consequence of the agonal waves per se, because of the inability to attenuate contraction-band formation even in the complete absence of these Ca^2+^ waves following myocyte treatment with ryanodine and thapsigargin. It is worth noting that although the waves were abolished in these treatments, [Ca^2+^]_i_ overload still occurred, which may indicate the maintenance of the cross-bridge interactions between actin and myosin needed to produce contraction bands. The addition of the calpain inhibitor MDL28170 precluded the saponin-induced derangements of the regular alignment of the Z-disc composed of the actin-binding protein α-actinin, although this agent still failed to attenuate the aggregation of actin fibres. This finding indicates that calpain contributes to the degradation of the myocyte structure, leading to contraction-band formation. In this context, among various structural proteins of cardiomyocytes α-actinin is reportedly the least susceptible to proteolysis by calpain^36^.

We revealed that the production of saponin-induced contraction bands was attenuated by pretreatment with BDM. This may be because of BDM’s inhibitory effects on myosin ATPase^20, 21^ and on the responsiveness of myofilaments to Ca^2+^^22^, which attenuate its cross-bridging functions. Our observations are in good agreement with previous experimental demonstrations that BDM can ameliorate contraction-band necrosis in the infarct border zone following ischaemia–reperfusion injury^37, 38^. In addition, we found that BDM augments myocyte Ca^2+^ cycling and thereby accelerates the frequency and the decay phase of the Ca^2+^ waves (Supplementary Fig. 7). These alterations may be explained by the preservation of ATP content following attenuated cross-bridge cycling^22, 23^ and dephosphorylation of various contractile proteins^39^ following BDM exposure. We also found that prior inhibition of the mitochondrial electron coupling pathway by FCCP attenuated saponin-induced agonal waves and the production of contraction bands. We assume that FCCP should inhibit mitochondrial ATP production and thereby attenuate the cross-bridge interactions required for contraction band formation.

**Figure 8:**
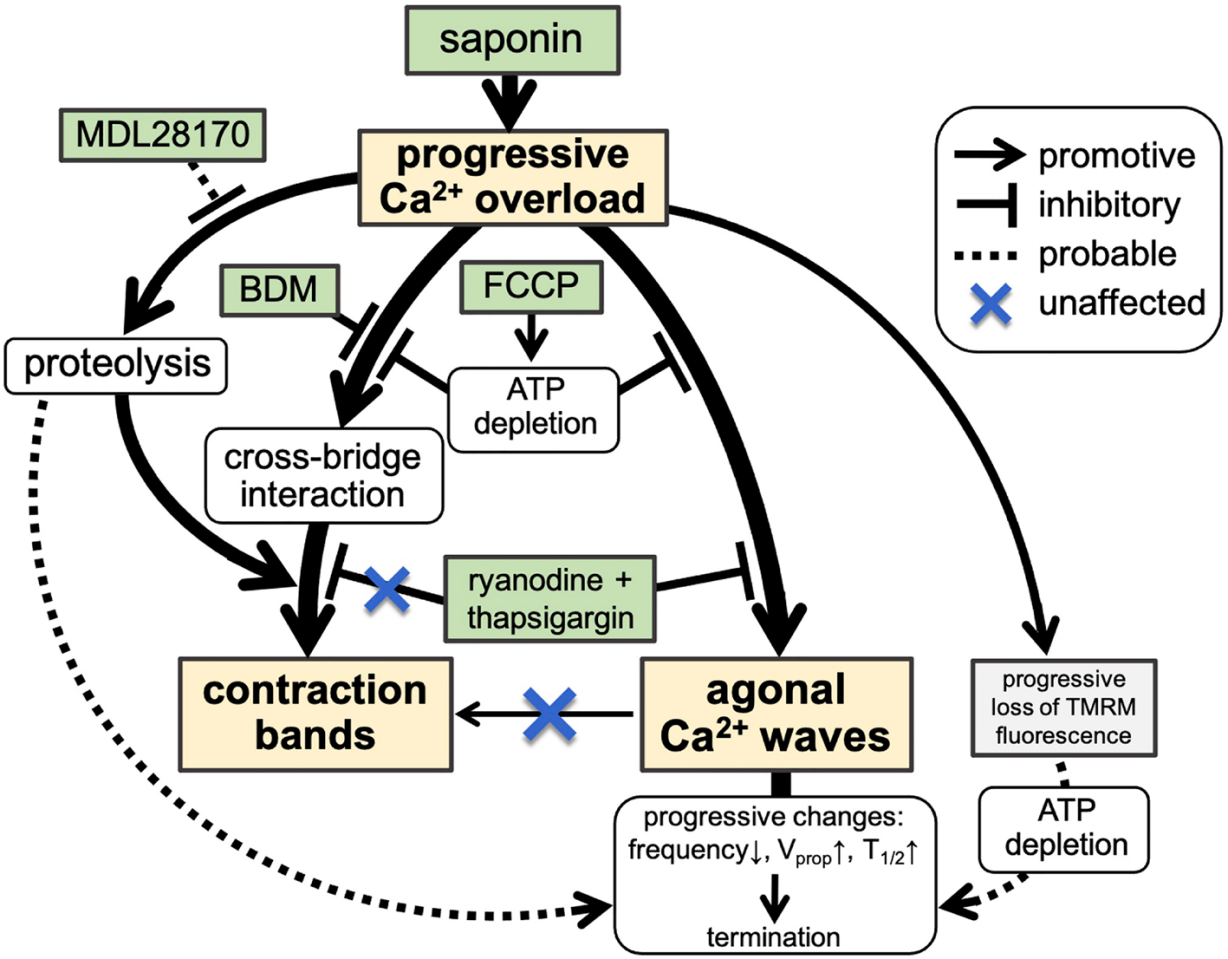
Schematic presentation of the saponin-induced generation of Ca^2+^ waves and contraction bands with effects of various pharmacological interventions.

Thus, we can summarise our findings as follows: saponin-induced [Ca^2+^]_i_ overload provokes agonal Ca^2+^ waves, and the coincident activation of Ca^2+^-dependent proteolysis and cross-bridge interaction are a prerequisite for the creation of contraction bands (Fig. 8). This is in good agreement with the idea that contraction bands arise locally within the reperfused area of the ischaemic heart, especially in the infarct border zone, where the myocardium preserves ATP^40, 41^ and the cross-bridge interactions. Accordingly, we can assume that for the formation of the contraction bands, the affected myocytes require energetically viable conditions, e.g., following reperfusion after short-term ischaemia, whereas myocytes in a low-energy state, e.g., during prolonged ischaemia, would undergo necrotic cell death^42, 43^. Therefore, we believe that the myocytes responsible for creating contraction bands following reperfusion after a short-term ischaemic insult may well be identifiable by their production of agonal waves, whereas the irreversibly energetically compromised myocardium undergoing coagulative necrosis would not produce any agonal waves as observed under pretreatment with FCCP (Fig. 7).

Despite the clarity of our results, we must consider some of the limitations of this study. First, this injured heart model was created using artificial membrane permeabilisation under excised, blood-free perfusion, and would not necessarily reflect the real pathophysiological setting of irreversible injury induced e.g., by ischaemia and reperfusion, and the pathological mechanisms for the latter model are more complex than those for the saponin model. However, since saponin overloads the myocyte [Ca^2+^]_i_ and disrupts myocyte integrity under intact conditions, i.e., energetically viable conditions of cardiomyocytes, we believe this chemical injury model would imitate the specific pathological conditions produced by reperfusion after short-term ischaemia in that the injured myocytes exhibit contraction band necrosis, a morphological change characteristic of this type of injury. It remains to be determined to what extent agonal waves arise in the irreversibly injured heart in situ. Second, it is still not clear how [Ca^2+^]_i_ overload contributes to the formation of contraction bands in injured myocytes: we only found that mechanical arrest can prevent their formation. Our data also suggest that the inhibition of calpain partially attenuated the derangements of cell structure, i.e., disruption of the alignment of the z-line composed of α-actinin. Further studies are needed to address the mechanisms underlying these outcomes including the identification of the saponin-induced degradation molecules that contribute to the connection between actin filaments and the sarcolemmal membrane, e.g., the membrane-spanning protein dystrophin-glycoprotein complex^44, 45^.

In conclusion, irreversible myocyte injury by saponin exposure provokes agonal Ca^2+^ waves and the formation of contraction bands following progressive [Ca^2+^]_i_ overload in the perfused heart. Our observations provide deeper insights into the mechanisms underlying contraction-band necrosis in the heart following ischaemia–reperfusion injury.

## Methods

### Materials and preparation procedures

This study was performed in accordance with the ARRIVE (Animal Research: Reporting of In Vivo Experiments) guidelines 2.0 (https://arriveguidelines.org). All of the animal experiments described in this study were conducted between December 2020 and August 2022 in accordance with *the Guide for the Care and Use of Laboratory Animals* (8th edition, National Academies Press, Washington DC, 2011) following approval by the Animal Research Committee at Kyoto Prefectural University of Medicine (approval No: M2020-219). Our experiments used male Wistar rats (9–12 weeks old) weighing 200–300 g (n = 57 hearts). These animals were placed under deep general anaesthesia using intraperitoneal injection with 0.1 mg/kg medetomidine, 3.0 mg/kg midazolam, and 5.0 mg/kg butorphanol, and then injected with heparin (2 U/g body weight) via the inferior vena cava before their hearts were quickly removed and then perfused using the Langendorff protocol and HEPES-buffered Tyrode’s solution supplemented with 137 mM NaCl, 4 mM KCl, 1 mM MgCl_2_, 0.33 mM NaH_2_PO_4_, 1.2 mM CaCl_2_, 10 mM HEPES and 10 mM glucose (pH = 7.4, adjusted by NaOH) for approximately 3 min. Once the blood was removed each heart was loaded with the Ca^2+^-indicator fluo4-AM (5.5 μM, Dojindo, Kumamoto, Japan) for 15 min at room temperature (23–25 °C) in accordance with the efficient conditions for fluo4 loading as previously described^8, 9^. Then the heart was perfused with Tyrode’s solution containing probenecid (0.1 mg/ml) for 5 min at 37 °C to augment the activity of esterase for de-esterification of the acetoxy methyl ester (AM) form of fluo4. The fluo4-loaded heart was then used in all subsequent experiments and was maintained under constant perfusion with HEPES-buffered, 0.3-mM Ca^2+^-containing Tyrode’s solution (5 mL/min) by a microtube pump (Chuo Rika, Japan) at 30 °C using a tube heater (Kawai Corporation, Japan) for efficient visualisation of [Ca^2+^]_i_ dynamics based on our experiences: higher temperatures up to 37 °C show a tendency to diminish the fluo4 fluorescence intensity. Organ ECGs were recorded using silver wires (0.5-mm diameter) placed at the bottom of a customised chamber.

### Rapid-scanning confocal imaging

We collected a series of spatiotemporal images of the fluo4 fluorescence intensity (463 × 357 μm, 349 × 269 pixels, 100 frames/s) in the subepicardial myocardium of the heart using constant Langendorff perfusion with 0.3 mM Ca^2+^ Tyrode’s solution before and during the application of saponin (0.4%). These images were obtained from the subepicardial myocardium of the fluo4-loaded rat hearts and high-speed, multipoint-scanning confocal microscopy facilitated by an upright microscope (BX-50WI, Olympus, Japan) and a spinning disc-type confocal unit CSU-21 (Yokogawa, Japan) under a glass coverslip (170-μm thickness) (n = 19 hearts). We prevented “coverslip hypoxia”^46^ of the heart by carefully placing the coverslip to the heart and limiting the observation period for each image to 30 s before the coverslip was removed, and the tissues were allowed to perfuse normally between imaging runs. The fluo4-fluorescence signals were detected using an image intensifier (C8600, Hamamatsu Photonics, Japan) and a charge-coupled device (CCD) camera (MiCAM02, Brainvision, Japan). All images were captured using a 20× objective lens (UMPLan FI, NA = 0.5, Olympus, Japan), and some experiments included electrical stimulation of the left ventricular apex using silver wires (0.5 mm in diameter). We also obtained a series of confocal images of tetramethylrhodamine methyl ester (TMRM) (Thermo Fisher Scientific, USA) fluorescence in the subepicardial myocardium using rapid-scanning confocal microscopy with excitation and emission wavelengths of 563 nm and > 665 nm, respectively. TMRM (100 nM) loading of the heart was performed by 15-min perfusion at room temperature (23–25 °C) followed by washing for 5 min prior to imaging.

### Chemicals

Saponin (MP Biomedicals, USA) was dissolved in Tyrode’s solution with adequate ultrasonic agitation for 30 min. In addition, several experiments required the hearts to be perfused with Tyrode’s solution supplemented with various reagents including ryanodine (FUJIFILM Wako Pure Chemical Corporation, Japan), thapsigargin (FUJIFILM Wako Pure Chemical Corporation, Japan), MDL28170 (Sigma‒Aldrich, USA), 2,3-butanedione monoxime (BDM) (Nacalai Tesque, Japan) or carbonyl cyanide 4-trifluoromethoxy phenylhydrazone (FCCP) (Abcam, UK), for specific pharmacological interventions.

### Confocal histochemical procedures

For confocal fluorescence imaging of the structural proteins for myocyte morphology, the hearts were fixed using Langendorff perfusion with 2% paraformaldehyde for 30 min (n = 29 hearts). We used Alexa Fluor 633 phalloidin (dilution 1:400, Life Technologies) for actin histochemistry. For sarcomeric α-actinin immunohistochemistry, incubation with a primary antibody (dilution 1:500, mouse monoclonal IgG, Sigma‒Aldrich, USA) for 48 h at 4 °C was followed by incubation with a secondary antibody for 48 h (dilution 1:500, Alexa Fluor 488, anti-mouse, Thermo Fisher Scientific, USA) prior to visualisation at room temperature. Confocal fluorescence images of both phalloidin and α-actinin were obtained using samples from the subepicardial surface of the heart using an FV-1000 confocal microscope (Olympus, Japan). The excitation wavelengths for phalloidin and sarcomeric α-actinin were 633 nm and 488 nm, respectively, and the emission wavelengths were > 650 nm and between 500 and 600 nm, respectively. To confirm membrane permeabilisation by saponin, we perfused in advance the membrane dye di-4-ANEPPS at 5 μM (Wako Pure Chemicals, Japan) and the membrane-impermeable DNA dye propidium iodide (PI) at 7.5 μM (Dojindo, Japan) for 5 min and subsequent washout for another 5 min for confocal imaging (n = 3 hearts).

### Quantitative data analysis

The fluorescence images were converted to multitiff files and analysed using ImageJ software (National Institutes of Health, USA). X-t images were obtained by plotting the fluorescence intensity along the longitudinal axis of each myocyte in the X–Y image movies and the propagation velocity (V_prop_) of the Ca^2+^ waves was calculated from the slope of the X-t image (Supplementary Fig. 1a,b). The half-decay time (T_1/2_) of the Ca^2+^ wave was obtained from the plot profiles of the X-t images (Supplementary Fig. 1c). Changes in the TMRM fluorescence intensity were obtained via the sequential evaluation of the total myocardium in each X–Y image. The quantitative data are presented as the mean ± standard deviation (SD). We analysed the data nonparametrically using the Kruskal‒Wallis test followed by the Steel‒Dwass test or the Mann‒Whitney U test via EZR (Saitama Medical Center, Jichi Medical University, Japan), a graphical user interface for R (The R Foundation for Statical Computing, Vienna, Austria)^47^. *P* values of < 0.05 were considered statistically significant.

## Supplementary Information

Supplementary Figures.

Supplementary Video 1.

Supplementary Video 2.

## Data Availability

All the data in this paper are available from the corresponding authors upon request.
