# Supplementary material for: Generation of myocyte agonal Ca2+ waves and contraction bands in perfused rat hearts following irreversible membrane permeabilisation
Source: Sci Rep. 2023 Jan 16;13:803. doi: 10.1038/s41598-023-27807-w (PMC9842683; doi:10.1038/s41598-023-27807-w)
Supplement: Supplementary file 1 — Supplementary Figures. [file 41598_2023_27807_MOESM1_ESM.pdf]

Supplementary Information

Generation of myocyte agonal Ca^{2+} waves and contraction bands in perfused rat hearts following irreversible membrane permeabilisation

Yuma Morishita, Shoko Tamura, Kentaro Mochizuki, Yoshinori Harada,
Tetsuro Takamatsu, Hajime Hosoi, Hideo Tanaka

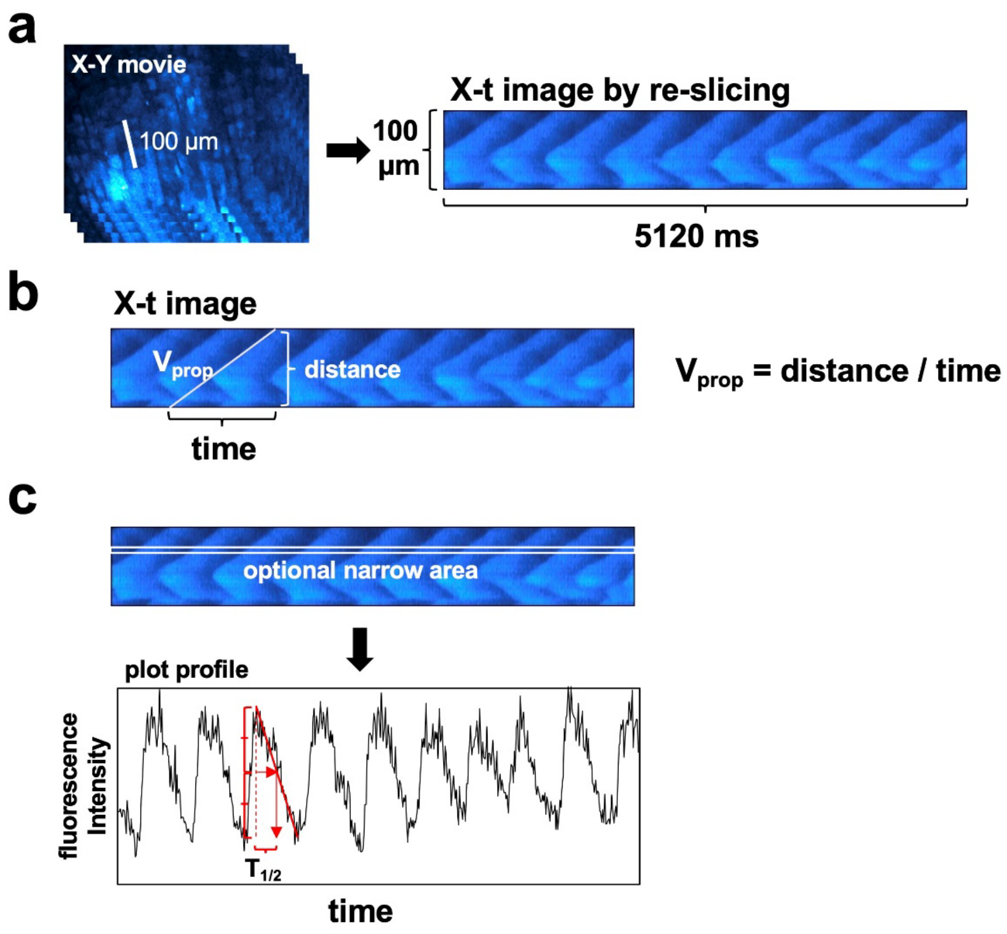

Supplementary Figure 1. Methods used to analyse Ca^{2+} waves. Creation of X-t images from X-Y movies **(a)**, measurement of propagation velocity (V_{prop}) of Ca^{2+} waves from X-t images **(b)**, and presentation and measurement of $T_{1/2}$ of fluorescence profiles from X-t images **(c)**.

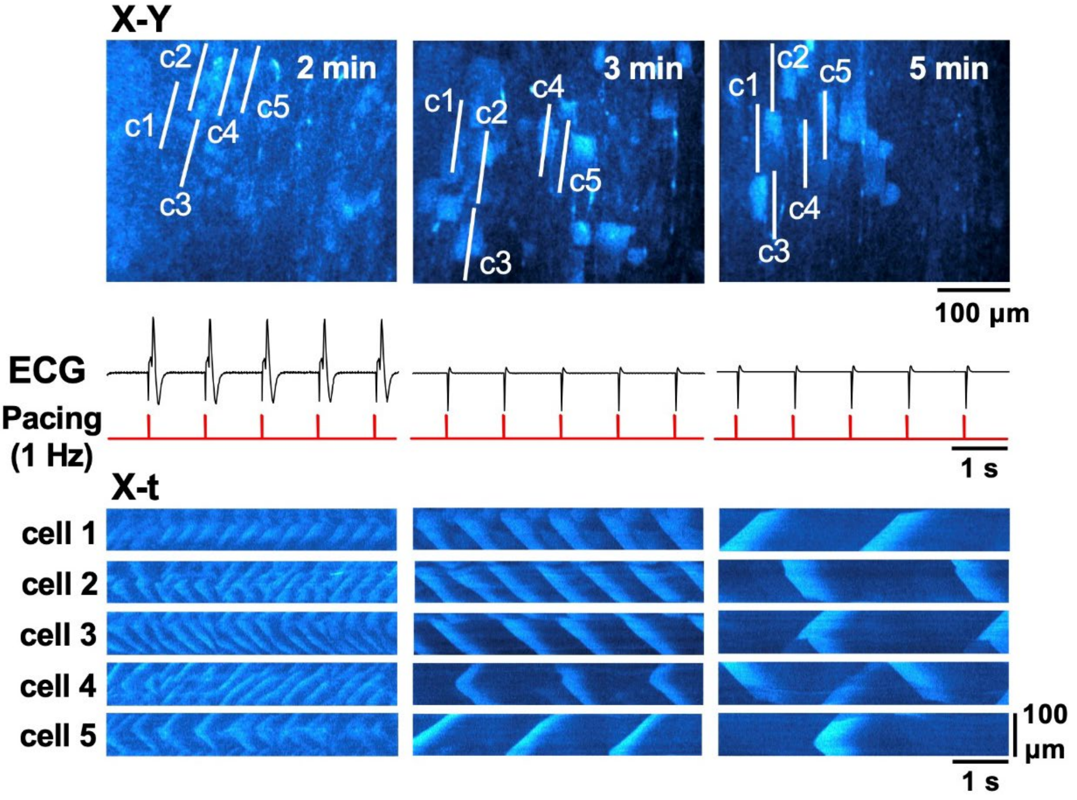

Supplementary Figure 2. Saponin-induced high-frequency Ca^{2+} waves were not affected by electrical stimulation of the heart. X-Y images (top panel) and corresponding X-t images (bottom panel) scanned along cells 1 - 5 (100 μm) in the X-Y images over a 5 min period. The corresponding electrocardiogram and pacing spikes (1-s pacing cycle length) are shown in the middle panel. Note that at 2 min after saponin application, the ECG exhibits a definitive QRS complex indicating ventricular excitation, whereas no QRS is identified at 3 or 5 min.

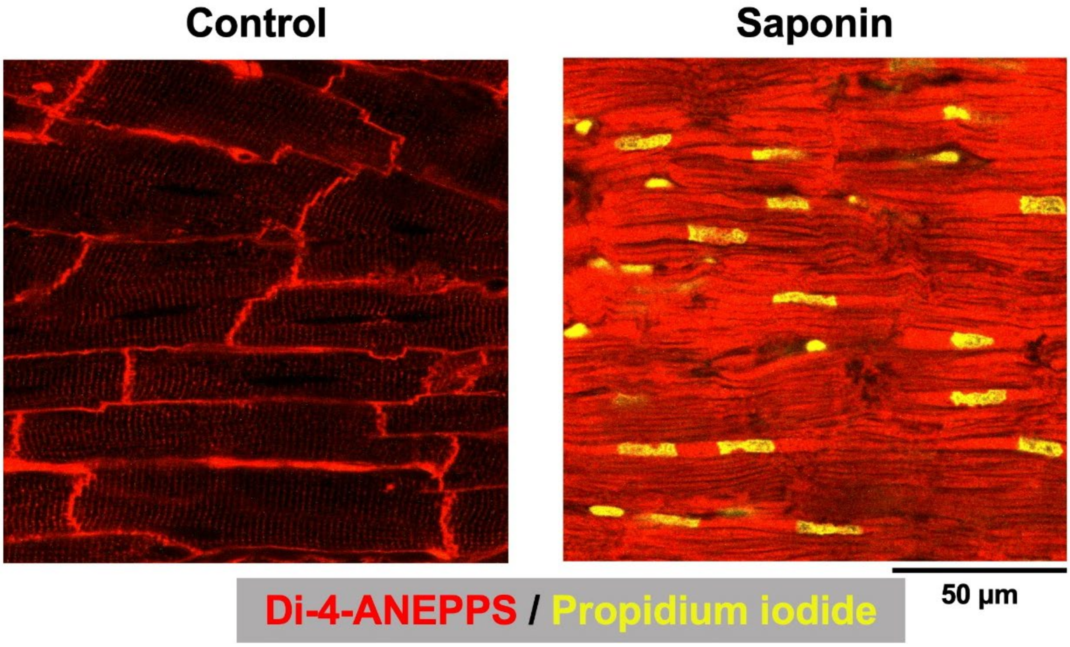

Supplementary Figure 3 Confirmation of the saponin-induced membrane permeabilisation of the myocardium. Confocal fluorescence images of membrane dye di-4-ANEPPS and nuclear dye propidium iodide (PI) of the heart in the absence of saponin (left) and 5 min after saponin perfusion (right).

a

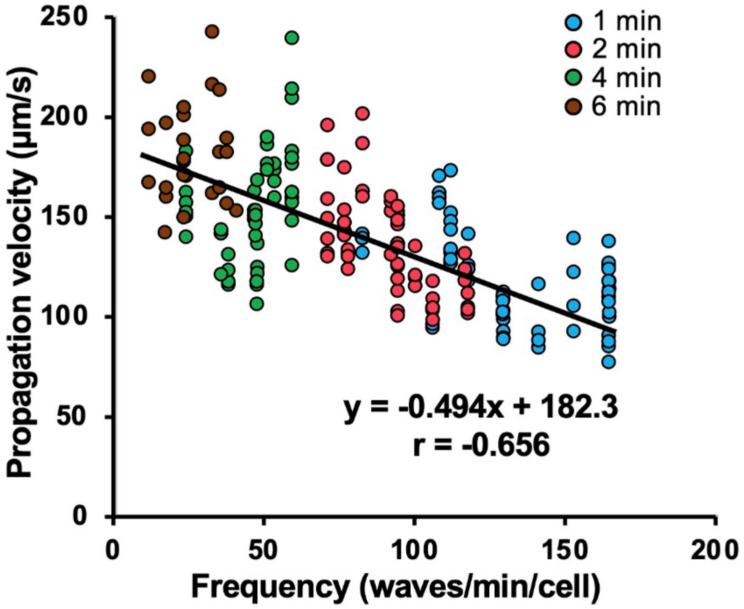

b

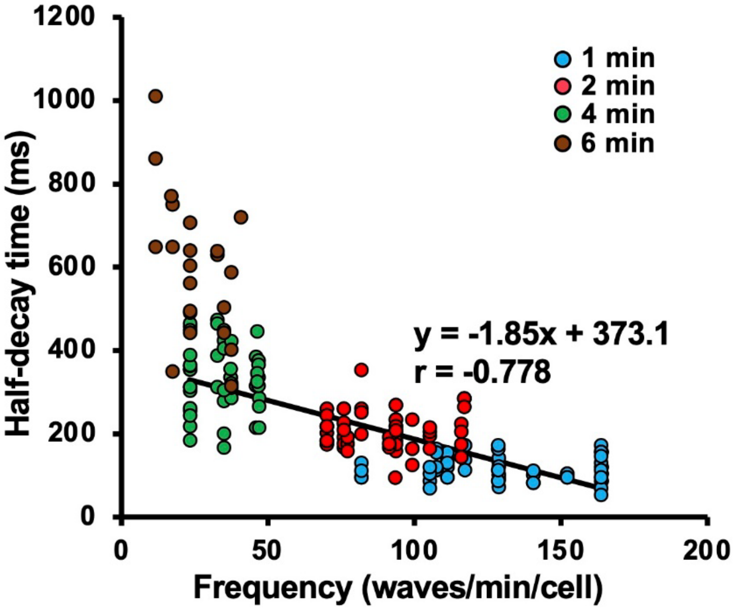

Supplementary Figure 4. a. Correlation between the frequency and propagation velocity (V_{prop}) of the Ca^{2+} waves at 1, 2, 4, and 6 min after saponin addition. **b.** Correlation between the frequency and half-decay time ($T_{1/2}$) of the Ca^{2+} waves at 1, 2, and 4 min after saponin addition.

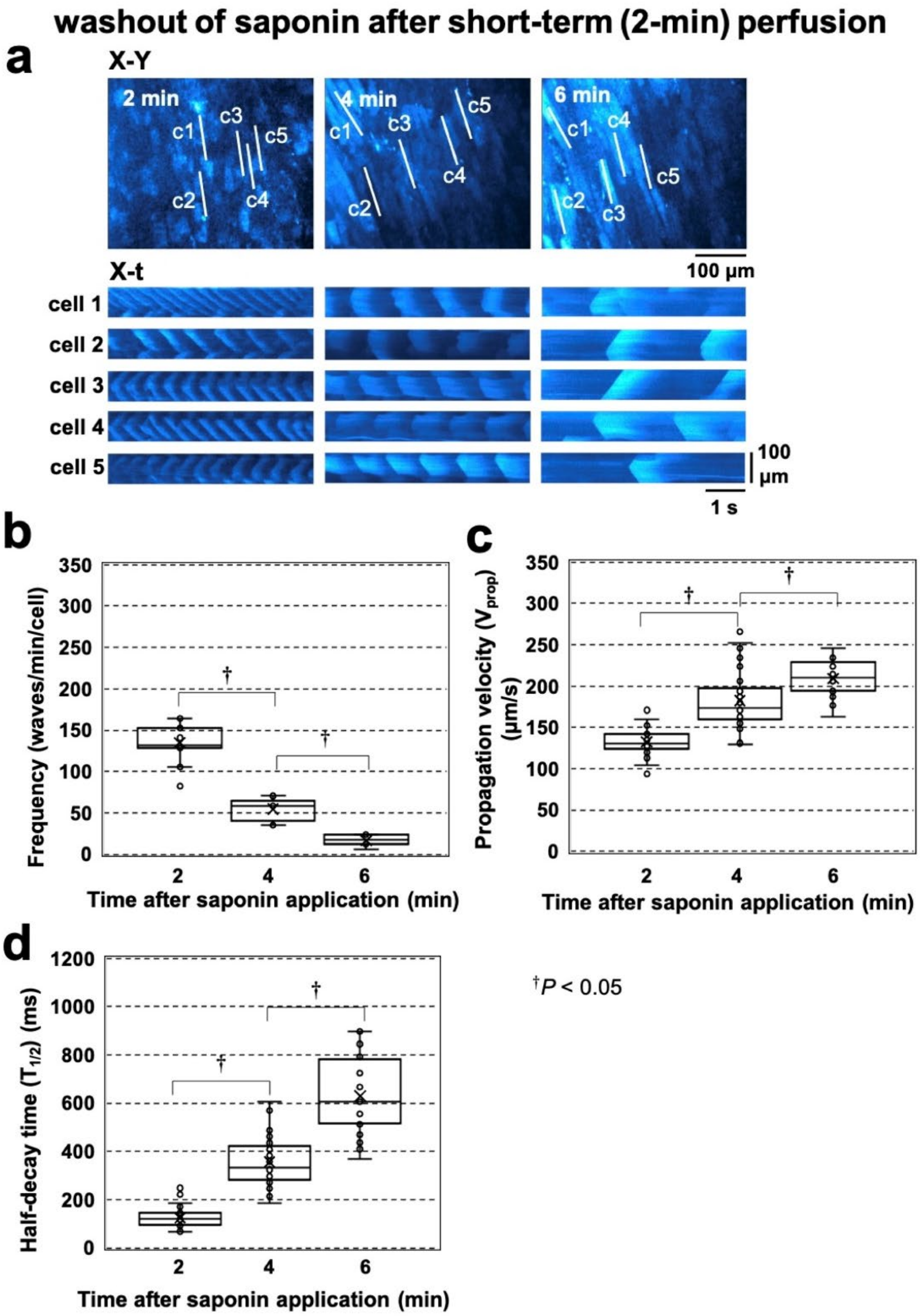

Supplementary Figure 5. Progressive changes in Ca^{2+} waves induced by short-term application of saponin for 2 min. X-Y fluo4-fluorescence images (upper panel) and corresponding X-t images (lower panel) scanned along the 100-µm c1 – 5 lines in five different myocytes (cells 1 - 5) in each of the X-Y images. Sequential box plots for frequency (**b**), V_{prop} (**c**), and $T_{1/2}$ (**d**) of the saponin-induced Ca^{2+} waves.

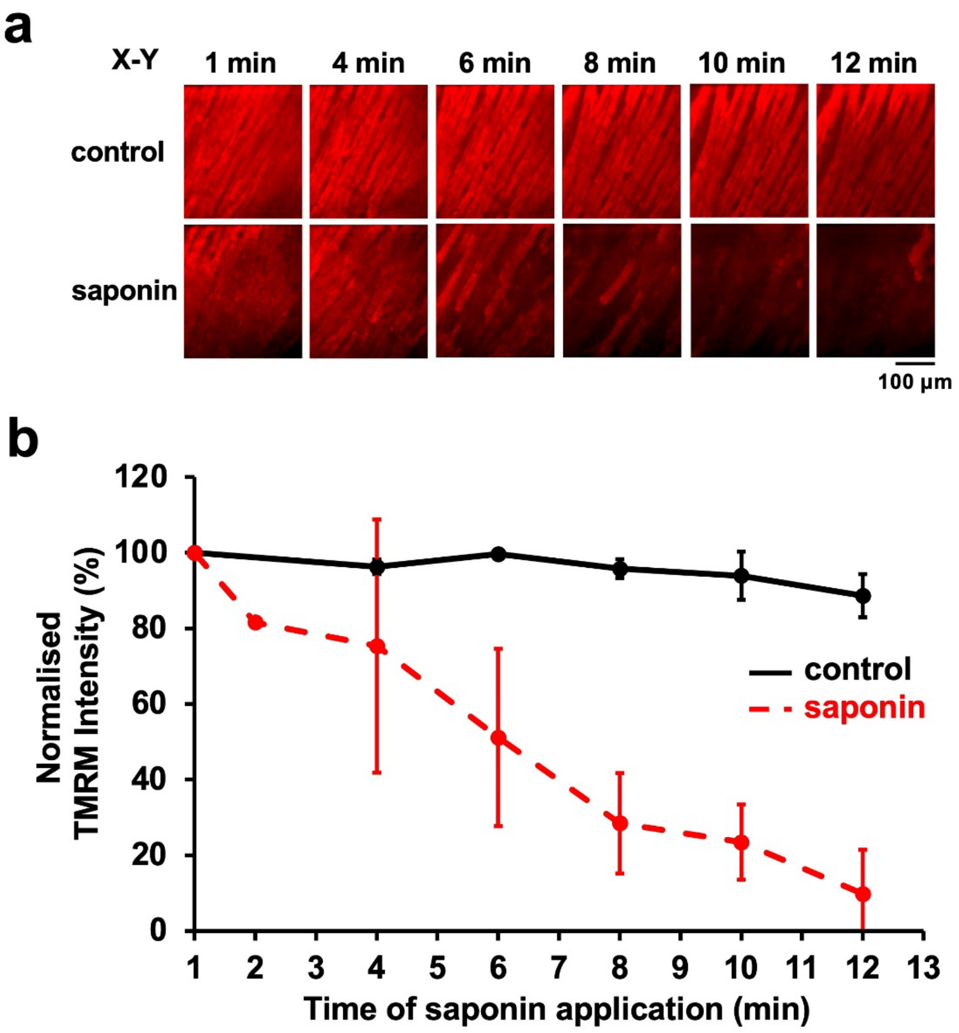

Supplementary Figure 6. Sequential changes in TMRM fluorescence intensity in the subepicardial myocardium following saponin treatment. **a.** Sequential changes in the X-Y fluorescence of TMRM with (lower) and without (upper) saponin treatment. Note that the fluorescence intensity of the individual myocytes diminished with time in the saponin-treated myocardium. **b.** Sequential changes in the averaged TMRM fluorescence intensity in each sample. Each averaged intensity was calculated from the X-Y image shown in (a) and normalised against the overall intensity at 1 min. Black solid circles with solid lines correspond to the control (obtained from 3 hearts), while red solid circles with dashed lines indicate the saponin-treated tissues (from 3 hearts).

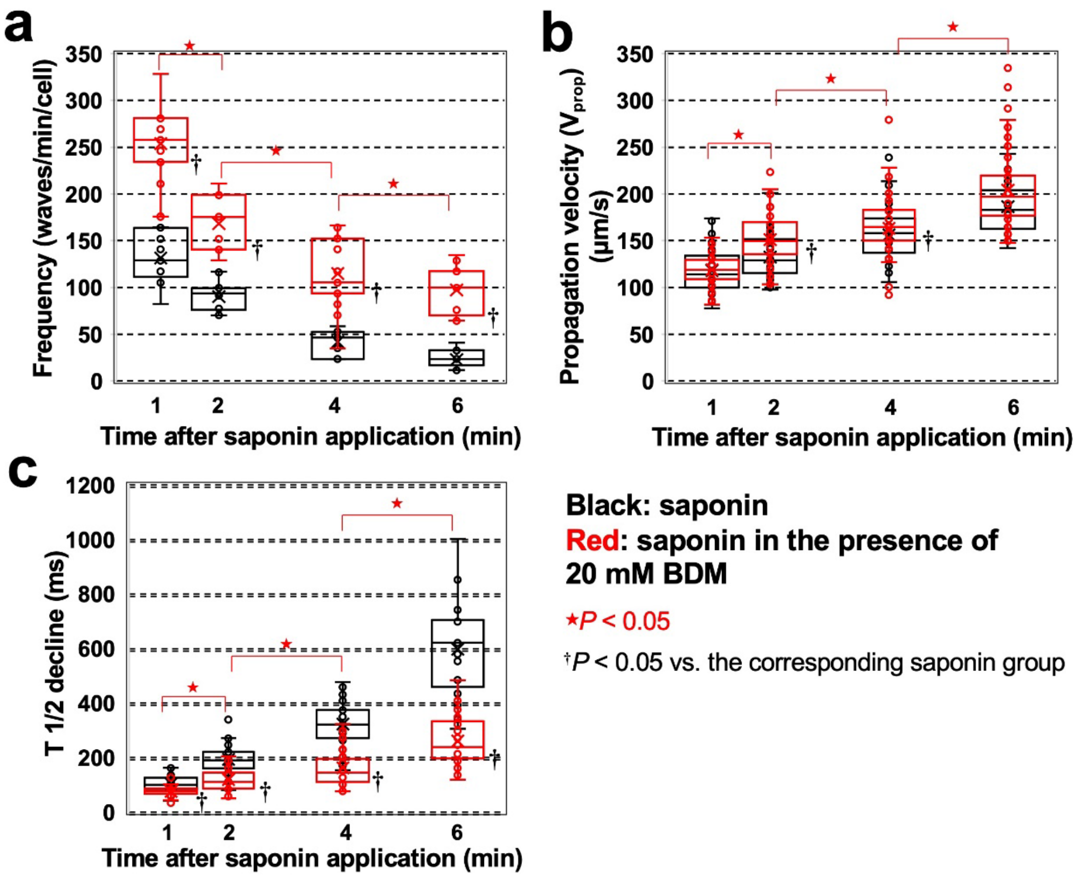

Supplementary Figure 7. Sequential box plots for frequency (**b**), V_{prop} (**c**), and $T_{1/2}$ (**d**) of saponin-induced Ca^{2+} waves with (**red**) and without (**black**) BDM. The frequency of the Ca^{2+} waves with BDM decreased from 253.7 ± 41.4 waves/min/cell at 1 min of exposure to 97.1 ± 25.5 waves/min/cell at 6 min post induction (60 cells). The V_{prop} value for the waves increased with time from 118.7 ± 16.9 μm/s at 1 min post exposure to 203.8 ± 39.2 μm/s at 6 min post exposure (239 waves). The $T_{1/2}$ was prolonged from 76.6 ± 17.8 ms at 1 min of exposure to 260.6 ± 83.6 ms at 6 min post induction (239 waves). ★ denotes $P < 0.05$ using the Kruskal-Wallis test followed by the Steel-Dwass test. † denotes $P < 0.05$ using the Mann-Whitney U test between the saponin plus BDM group and the corresponding saponin group.

Legends for Supplementary Videos

Supplementary Video 1. Effects of saponin on $[Ca^{2+}]_i$ dynamics of the subepicardial myocardium of the Langendorff-perfused rat heart.

In the absence of saponin (control), the individual myocytes exhibit spatiotemporally uniform Ca^{2+} transients during systole (left panel). In contrast, 1 min after commencement of saponin perfusion the myocytes exhibit highly frequent, repetitive Ca^{2+} waves occurring asynchronously among the myocytes (right panel).

Supplementary Video 2. Sequential changes in $[Ca^{2+}]_i$ dynamics by saponin perfusion.

The myocytes exhibit a gradual decrease in the frequency of the Ca^{2+} waves 2 min (**a**), 4 min (**b**), and 6 min (**c**) after commencement of saponin perfusion and show high, static fluo4-fluorescence intensity in 10 min (**d**).
